# Short-Term Stability of Serum and Liver Extracts for Untargeted Metabolomics and Lipidomics

**DOI:** 10.3390/antiox12050986

**Published:** 2023-04-24

**Authors:** Jiri Hricko, Lucie Rudl Kulhava, Michaela Paucova, Michaela Novakova, Ondrej Kuda, Oliver Fiehn, Tomas Cajka

**Affiliations:** 1 Institute of Physiology of the Czech Academy of Sciences, Videnska 1083, 14200 Prague, Czech Republic; jiri.hricko@fgu.cas.cz (J.H.); lucie.kulhava@fgu.cas.cz (L.R.K.); michaela.paucova@fgu.cas.cz (M.P.); michaela.novakova@fgu.cas.cz (M.N.); ondrej.kuda@fgu.cas.cz (O.K.); 2 West Coast Metabolomics Center, University of California, 451 Health Sciences Drive, Davis, CA 95616, USA; ofiehn@ucdavis.edu

**Keywords:** metabolomics, lipidomics, stability, shipping, oxidation, serum, liver, tissue, liquid chromatography, mass spectrometry, LC-MS

## Abstract

Thermal reactions can significantly alter the metabolomic and lipidomic content of biofluids and tissues during storage. In this study, we investigated the stability of polar metabolites and complex lipids in dry human serum and mouse liver extracts over a three-day period under various temperature conditions. Specifically, we tested temperatures of −80 °C (freezer), −24 °C (freezer), −0.5 °C (polystyrene box with gel-based ice packs), +5 °C (refrigerator), +23 °C (laboratory, room temperature), and +30 °C (thermostat) to simulate the time between sample extraction and analysis, shipping dry extracts to different labs as an alternative to dry ice, and document the impact of higher temperatures on sample integrity. The extracts were analyzed using five fast liquid chromatography-mass spectrometry (LC-MS) methods to screen polar metabolites and complex lipids, and over 600 metabolites were annotated in serum and liver extracts. We found that storing dry extracts at −24 °C and partially at −0.5 °C provided comparable results to −80 °C (reference condition). However, increasing the storage temperatures led to significant changes in oxidized triacylglycerols, phospholipids, and fatty acids within three days. Polar metabolites were mainly affected at storage temperatures of +23 °C and +30 °C.

## 1. Introduction

Metabolomics and lipidomics provide comprehensive views of numerous small molecules in biological samples such as biofluids, tissues, and cells [1]. However, both approaches are sensitive to variability in sample handling and preparation [2]. Over the last decade, many studies have examined factors affecting metabolite stability, mainly in biofluids such as plasma, serum, and urine [3], since these matrices are a convenient specimen type for investigation. For plasma and serum, these factors included (i) sample-related factors (e.g., collection time [4], hemolysis [5], tube additives [6], fasting status [7]), (ii) sample processing (e.g., centrifugation conditions [8], time delay and temperature [9]), (iii) post-processing (e.g., time delay temperature [10]), and (iv) sample storage (e.g., freeze/thaw cycles [10], storage time [5]). Additional sample processing factors were studied for urine, such as osmolarity, sample volume [11], and filtration [12]. However, biofluids contain metabolites exported from different organs, which complicates data interpretation [13]. Thus, metabolomic and lipidomic profiling of tissues provides more profound insights into metabolism than biofluids. While the liver, brain, and heart are the top three mammalian organs reported for metabolomics in pre-clinical animal studies [14], stability studies on tissues are rarely discussed [15].

Most stability studies are usually conducted with the collected matrix [3], but much less is known about the stability of isolated metabolites in dry extracts. Here, we evaluated the short-term stability (three days) of polar metabolites and complex lipids in dry human serum and mouse liver extracts under different temperature conditions, intending to simulate the time between sample extraction and analysis using a multiplatform liquid chromatography-mass spectrometry (LC-MS)-based approach [16]. These data can also be useful for laboratories conducting sample extraction on one side and running the instrumental platforms on the other, which may require shipping the dry extracts. Finally, we provide documentation on how higher temperatures can compromise the sample integrity, which can be used to train laboratory personnel.

## 2. Materials and Methods

### 2.1. Materials and Reagents

LC-MS-grade solvents, including acetonitrile, isopropanol, methanol, and water, as well as methyl *tert*-butyl ether (MTBE) and LC-MS-grade mobile phase modifiers, such as ammonium formate, ammonium acetate, formic acid, and acetic acid, were from J.T.Baker, Merck, and VWR International (Prague, Czech Republic). The internal standards were obtained from Cambridge Isotope Laboratories (Tewksbury, MA, USA), Cayman Chemical (Tallinn, Estonia), and Merck. Standards of PC 16:0/18:1, PC 18:1/16:0, PC 16:0/18:2, and PC 18:0/20:4 were from Merck.

Human serum (S7023-100ML) was obtained from Merck. Mouse liver (C57BL/6J) was obtained from the Institute of Physiology of the Czech Academy of Sciences.

### 2.2. Sample Preparation

Metabolomic and lipidomic profiling of human serum and mouse liver was conducted using a workflow for the lipidome, metabolome, and exposome analysis (LIMeX) [16,17]. A biphasic solvent extraction using methanol, MTBE, and water was used for sample extraction [18], with some modifications given below.

#### 2.2.1. Serum

An aliquot of 25 µL serum sample was mixed with 765 μL of ice-cold methanol/MTBE mixture (165 µL methanol and 600 µL MTBE, respectively) containing internal standards (methanol: CAR 14:0-*d*_9_, CAR 16:0-*d*_3_, CAR 18:0-*d*_3_, Cer d18:1/17:0, cholesterol-*d*_7_, CL 16:0/16:0/16:0/16:0, DG 12:0/12:0/0:0, DG 18:1/0:0/18:1-*d*_5_, DG 18:1/2:0/0:0, Hex-Cer d18:1/17:0, LPC 17:1, LPE 17:1, LPG 17:1, LPS 17:1, MG 17:0/0:0/0:0, oleic acid-*d*_9_, PC 15:0/18:1-*d*_7_, PE 17:0/17:0, PG 17:0/17:0, PI 15:0/18:1-*d*_7_, PS 17:0/17:0, SM d18:1/17:0, sphingosine d17:1, TG 17:0/17:1/17:0-*d*_5_, TG 20:0/20:1/20:0-*d*_5_; MTBE: CE 22:1) and this mixture was shaken (30 s). Then, 165 µL of 10% methanol with internal standards (3-hydroxybutyric acid-*d*_4_, acetylcholine-*d*_4_, alanine(^13^C_3_; ^15^N), arginine(^13^C_6_; ^15^N_4_), aspartic acid(^13^C_4_; ^15^N), betaine-*d*_9_, butyrobetaine-*d*_9_, caffeine-*d*_9_, CAR 2:0-*d*_3_, CAR 3:0-*d*_3_, CAR 4:0-*d*_3_, CAR 6:0-*d*_3_, CAR 8:0-*d*_3_, CAR 10:0-*d*_3_, CAR 12:0-*d*_9_, choline-*d*_9_, citrulline-*d*_4_, cotinine-*d*_3_, creatine-*d*_3_, creatinine-*d*_3_, cystine(^13^C_6_; ^15^N_2_), glucose-*d*_7_, glutamic acid(^13^C_5_; ^15^N), glycine(^13^C_2_; ^15^N), histidine(^13^C_6_; ^15^N_3_), isoleucine(^13^C_6_; ^15^N), leucine(^13^C_6_; ^15^N), lysine(^13^C_6_; ^15^N_2_), metformin-*d*_6_, methionine(^13^C_5_; ^15^N), *N*-methylnicotinamide-*d*_4_, ornithine-*d*_6_, phenylalanine(^13^C_9_; ^15^N), proline(^13^C_5_; ^15^N), serine(^13^C_3_; ^15^N), succinic acid-*d*_4_, threonine(^13^C_4_; 15N), trimethylamine *N*-oxide-*d*_9_, tyrosine(^13^C_9_; ^15^N), valine(^13^C_5_; ^15^N)) was added, vortexed (10 s), and centrifuged (16,000 rpm, 5 min, 4 °C). These internal standards showed minimal or no signal in non-spiked serum and liver extracts.

An aliquot of 70 µL of the bottom phase was collected and evaporated for the metabolomic analysis. The dry serum extracts were resuspended in 70 µL of an acetonitrile/water (4:1) mixture with two internal standards (12-[[(cyclohexylamino)carbonyl]amino]-dodecanoic acid (CUDA) and Val-Tyr-Val), shaken (30 s), centrifuged (16,000 rpm, 5 min, 4 °C), and analyzed using the HILIC metabolomics platforms [18]. Another 70 µL aliquot of the bottom phase was mixed with 210 µL of an isopropanol/acetonitrile (1:1) mixture, shaken (30 s), centrifuged (16,000 rpm, 5 min, 4 °C), and the supernatant was evaporated. The dry serum extracts were resuspended in 5% methanol/0.2% formic acid containing two internal standards (CUDA and Val-Tyr-Val), shaken (30 s), centrifuged (16,000 rpm, 5 min, 4 °C), and analyzed using the RPLC metabolomics platform [18].

For the lipidomic analysis, an aliquot of 100 µL of the upper phase was collected, evaporated, and the dry serum extracts were resuspended in 100 µL methanol containing an internal standard (CUDA), shaken (30 s), centrifuged (16,000 rpm, 5 min, 4 °C), and used for LC-MS analysis [18].

#### 2.2.2. Liver

An amount of 20 mg of liver samples was homogenized (1.5 min) with 275 μL of methanol containing internal standards (see Section 2.2.1) using a grinder. Then, 1 mL of MTBE with an internal standard (see Section 2.2.1) was added and shaken (30 s). Finally, 275 μL of 10% methanol with internal standards (see Section 2.2.1) was added, and after vortexing (10 s), the tubes were centrifuged (16,000 rpm, 5 min, 4 °C).

For the metabolomic analysis, the procedure was the same as for serum extracts (see Section 2.2.1).

For the lipidomic analysis, an aliquot of 100 µL of the upper phase was collected, evaporated, and the dry liver extracts were resuspended in 300 µL of methanol containing an internal standard (CUDA), shaken (30 s), centrifuged (16,000 rpm, 5 min, 4 °C), and used for LC-MS analysis [18].

### 2.3. Storage Conditions

The extracts (upper or bottom phase) from multiple extractions were combined for each matrix. Then, 70 µL of the combined polar extracts (metabolomics) were pipetted to 36 tubes. For complex lipids (lipidomics), 100 µL of the combined organic extracts were pipetted to 36 tubes. After solvent evaporation, each set of six tubes was stored for three days at particular storage conditions in a cardboard box for tubes: (i) −80 ± 0.2 °C (freezer), (ii) −24 ± 0.2 °C (freezer), (iii) −0.5 °C (median value) (polystyrene box with ice packs), (iv) +5 ± 1 °C (refrigerator), (v) +23 ± 0.2 °C (laboratory, room temperature), and (vi) +30 ± 0.1 °C (thermostat). After this period, the dry extracts were resuspended for a particular LC-MS platform and analyzed. The temperature was monitored using temperature data loggers (Comet System, Roznov pod Radhostem, Czech Republic). The inner size of a polystyrene box was 27 cm × 24 cm × 21 cm with a 5-cm wall thickness, containing multiple gel-based ice packs (GIO’STYLE, Urgnano, Italy) frozen over one day at −24 °C. The samples were kept in a paper storage box (13 cm × 13 cm × 5.2 cm).

### 2.4. LC-MS Conditions

The LC-MS system comprised a Vanquish UHPLC system (Thermo Fisher Scientific, Bremen, Germany), a heated electrospray ionization (HESI-II) probe (Thermo Fisher Scientific), and a Q Exactive Plus mass spectrometer (Thermo Fisher Scientific) [18].

#### 2.4.1. Untargeted Metabolomics

The ACQUITY Premier BEH Amide column (50 mm × 2.1 mm i.d.; 1.7 μm particle size) equipped with a VanGuard FIT cartridge (5 mm × 2.1 mm i.d.; 1.7 μm particle size) (Waters, Milford, MA, USA) was utilized to separate polar metabolites based on the HILIC mechanism. The separation was carried out at a flow rate of 0.8 mL/min with the column kept at a temperature of 45 °C. The mobile phase included (A) water with 10 mM ammonium formate and 0.125% formic acid and (B) acetonitrile/water (95:5) with 10 mM ammonium formate and 0.125% formic acid [18]. Separation was achieved through the following gradient: 0 min 100% (B); 0–0.5 min 100% (B); 0.5–2.0 min from 100% to 70% (B); 2.0–2.6 min from 70% to 30% (B); 2.6–3.2 min from 30% to 100% (B); 3.2–3.4 min 100% (B) +1 min preinjection steps. The injection volumes were 1 μL (serum) and 0.5 µL (liver) in ESI(+) and 5 µL (serum, liver) in ESI(−). The sample temperature was maintained at 4 °C.

The ACQUITY Premier HSS T3 column (50 mm × 2.1 mm i.d.; 1.8 μm particle size) equipped with a VanGuard FIT cartridge (5 mm × 2.1 mm i.d.; 1.8 μm particle size) (Waters) was utilized to separate polar metabolites based on the RPLC mechanism. The separation was carried out at a flow rate of 0.6 mL/min with the column kept at a temperature of 45 °C. The mobile phase included (A) water with 0.2% formic acid and (B) methanol with 0.1% formic acid [18]. Separation was achieved through the following gradient: 0 min 1% (B); 0–0.5 min 1% (B); 0.5–2 min from 1% to 60% (B); 2–2.3 min from 60% to 99% (B); 2.3–2.8 min 99% (B); 2.8–2.9 min from 99% to 1% (B); 2.9–3.4 min 1% (B) + 1 min preinjection steps. An injection volume of 5 μL was used. The sample temperature was kept at 4 °C.

The ion source parameters were as follows: sheath gas pressure, 60 arbitrary units; aux gas flow, 25 arbitrary units; sweep gas flow, 4 arbitrary units; capillary temperature, 300 °C; aux gas heater temperature, 475 °C; spray voltage: 3.5 kV for ESI(+), −3.0 kV for ESI(−). The mass spectrometer settings were set to MS1 mass range, *m*/*z* 60–900; MS1 resolving power, 35,000 FWHM (*m*/*z* 200); the number of data-dependent scans per cycle, 2; MS/MS resolving power, 17,500 FWHM (*m*/*z* 200). For MS/MS experiments, normalized collision energies of 20, 30, and 40% were set up for both polarities.

#### 2.4.2. Untargeted Lipidomics

The ACQUITY Premier BEH C18 column (50 mm × 2.1 mm i.d.; 1.7 μm particle size) equipped with a VanGuard FIT cartridge (5 mm × 2.1 mm i.d.; 1.7 μm particle size) (Waters) was utilized to separate complex lipids based on the RPLC mechanism. The separation was carried out at a flow rate of 0.8 mL/min with the column kept at a temperature of 65 °C. For LC-ESI(+)-MS lipidomic analysis, the mobile phase included (A) 60:40 acetonitrile/water with 10 mM ammonium formate and 0.1% formic acid, and (B) 90:10:0.1 isopropanol/acetonitrile/water with 10 mM ammonium formate and 0.1% formic acid. For LC-ESI(−)-MS lipidomic analysis, the mobile phase included (A) 60:40 acetonitrile/water with 10 mM ammonium acetate and 0.1% acetic acid, and (B) 90:10:0.1 isopropanol/acetonitrile/water with 10 mM ammonium acetate and 0.1% acetic acid [18]. Separation was achieved through the following gradient for LC-ESI(+)-MS: 0 min 15% (B); 0–0.5 min from 15% to 30% (B); 0.5–0.6 min from 30% to 50% (B); 0.6–2.8 min from 50% to 80% (B); 2.8–3.2 min from 80% to 99% (B); 3.2–3.4 min 99% (B); 3.4–3.5 min from 99% to 15% (B); 3.5–3.7 min 15% (B) + 1 min preinjection steps. For the LC-ESI(−)-MS, a slightly modified gradient was used: 0 min 15% (B); 0–0.5 min from 15% to 30% (B); 0.5–0.6 min from 30% to 50% (B); 0.6–2.4 min from 50% to 75% (B); 2.4–2.5 min from 75% to 99% (B); 2.5–2.9 min 99% (B); 2.9–3.0 min from 99% to 15% (B); 3.0–3.2 min 15% (B) + 1 min preinjection steps. The injection volumes were 1 μL (serum) and 0.2 µL (liver) in ESI(+) and 5 µL (serum, liver) in ESI(−). The sample temperature was kept at 4 °C.

The ion source parameters were as follows: sheath gas pressure, 60 arbitrary units; aux gas flow, 25 arbitrary units; sweep gas flow, 4 arbitrary units; capillary temperature, 300 °C; aux gas heater temperature, 475 °C; spray voltage: 3.5 kV for ESI(+), −3.0 kV for ESI(−). The mass spectrometer settings were set to MS1 mass range, *m*/*z* 200–1700; MS1 resolving power, 35,000 FWHM (*m*/*z* 200); the number of data-dependent scans per cycle, 2; MS/MS resolving power, 17,500 FWHM (*m*/*z* 200). For MS/MS experiments, a normalized collision energy of 20% was used in positive ion mode, and normalized collision energies of 10, 20, and 30% were set up in negative ion mode.

#### 2.4.3. Iterative MS/MS Acquisition

After the injection of the resuspension solvent for each platform, an initial exclusion list with *m*/*z* values for the full retention time range was generated. Next, the MS1 acquisition and data-dependent analysis (DDA)-MS/MS were conducted for the QC sample (a mixture of all study samples for each matrix). The ProteoWizard software [19] was utilized to generate an MS2 file (peak picking (vendor): level: 2-2; msLevel: 2-2; threshold: absolute 0.0001 most intense; output: MS2), which was then submitted to an R script [20]. An updated exclusion list was created, containing previously excluded *m*/*z* values (IE-1) and newly excluded *m*/*z* values (IE-2), which mostly belonged to high-abundant precursor ions of metabolites. During the run, MS/MS spectra were collected for high-abundant precursor ions for the QC samples and selected samples within each group (method IE-1), while MS/MS spectra for low-abundant precursor ions were acquired for the remaining samples within each group (method IE-2).

### 2.5. Quality Control

To ensure quality control, several measures were taken [13], including (i) randomization of the samples within the sequence, (ii) regular injection of quality control (QC) pool samples at the beginning, end, and between every ten actual samples for a specific matrix, (iii) analysis of method blanks, (iv) analysis of serial dilution samples prepared from QC sample (0, 1/16, 1/8, 1/4, 1/2, 1), and (v) control of the chromatographic peak shape, retention time, and intensity of internal standards.

### 2.6. Data Processing

The LC-MS instrumental files generated during metabolomic and lipidomic analyses were processed using MS-DIAL v. 4.9.221218 software [21] with the following parameters: (i) data collection: MS1 tolerance, 0.01; MS2 tolerance, 0.025; (ii) peak detection: minimum peak height: 15,000; mass slice width, 0.05; smoothing method, Linear Weighted Moving Average; smoothing level, 2; (iii) MS/MS identification setting: accurate mass tolerance (MS1), 0.005; accurate mass tolerance (MS2), 0.005; identification score cut off, 80% (lipidomics), 85% (metabolomics); (iv) alignment: retention time tolerance: 0.05 min; MS1 tolerance: 0.01 Da; peak count filter: 5%; gap filling by compulsion, true. Polar metabolites and exposome compounds were annotated based on retention time–*m*/*z* match from an in-house spectral library along with MS/MS libraries available from various sources (NIST20, MassBank.us, and MS-DIAL MS/MS library v. 15). Complex lipids were annotated using in silico MS/MS spectra available in MS-DIAL software.

Exported data sets for each matrix and platform as signal intensity from the detector (peak heights) were further filtered by removing metabolites with (i) a max sample peak height/blank peak height average < 10, (ii) an *R*^2^ < 0.8 from a dilution series of QC sample, and (iii) a relative standard deviation (RSD) > 20% from QC samples injected between 10 actual study samples. Data were then normalized using locally estimated scatterplot smoothing (LOESS) with QC samples injected between 10 actual study samples. The list of all annotated polar metabolites and complex lipids is available in Tables S1 and S2. Table S3 provides an overview of lipid classes from MS-DIAL used to annotate complex lipids in serum and liver extracts. Data from the ionization mode with higher annotation rates for lipids that ionized in both modes were used.

### 2.7. Statistical Analysis

First, principal component analysis (PCA) was performed in MetaboAnalyst 5.0 [22]. PCA was created separately for polar metabolites and complex lipids data sets (base 10 log-transformed and Pareto scaled).

Second, for each matrix, we assigned a value of 1 to the median peak height (*n* = 6) of a particular metabolite at −80 °C (reference condition). The peak heights in other groups were then proportionally calculated based on this value to provide normalized peak heights. Student’s *t*-tests were used to analyze peak intensity differences between −80 °C (reference condition) and all other storage temperatures. Bonferroni correction was used to reduce the probability of obtaining false-positive results (type I error) [23]. A *p*-value 0.05/602 = 8.31 × 10^–5^ was considered statistically significant for human serum extracts, and a *p*-value 0.05/724 = 6.91 × 10^–5^ was considered significant for mouse liver extracts. The median value of each metabolite was also calculated for each storage condition to assess the up (>1) or down (<1) trend.

## 3. Results and Discussion

**Figure 1:**
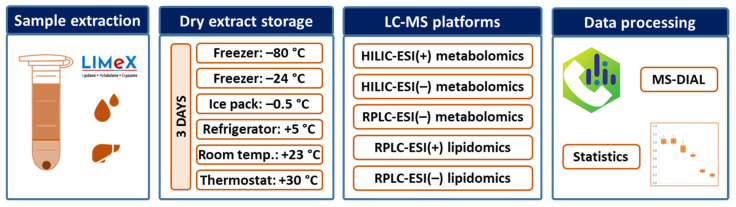
The experimental design of the study focused on the short-term stability of polar metabolites and lipids in dry human serum and mouse liver extracts under different temperature conditions.

We investigated the changes in metabolites in dry human serum and mouse liver extracts during short-term storage at different temperature conditions (Figure 1).

**Figure 2:**
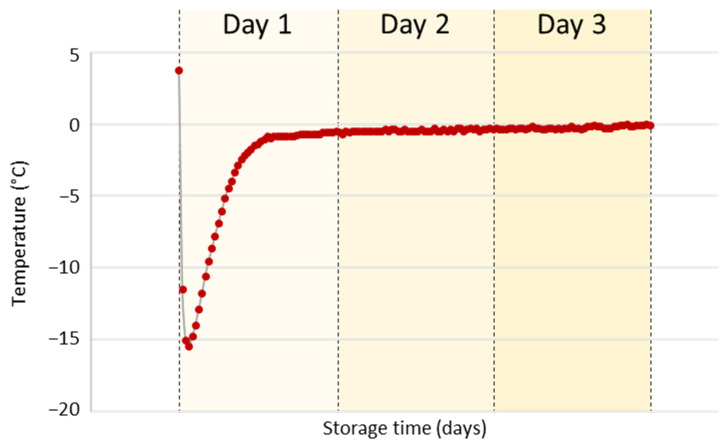
Temperature profile during storage of dry serum and liver extracts in a polystyrene box with gel-based ice packs within three days. The temperature was monitored using a data logger registering temperature every 30 min.

Separate fractions from a bi-phase extraction for metabolomics and lipidomics analysis were collected, evaporated, and stored for three days at various temperatures. A temperature of −80 °C in the freezer was used as the reference condition considered to induce minimal unwanted chemical reactions. Comparative storage conditions were a freezer set at −24 °C, a polystyrene box with gel-based ice packs maintained at −0.5 °C, a refrigerator set at +5 °C, laboratory environment maintained at +23 °C, and finally, a thermostat set at +30 °C. Lower temperature conditions (−24 °C) could occur for short time periods during routine sample handling while dry extracts were readied for LC-MS analysis. Lower temperatures (−24 °C, −0.5 °C) might also be used during transportation as an alternative to dry ice, which poses a safety hazard and is hampered by high costs. Some global shipping companies even decline to transport such items or do not replenish dry ice while in transit. A constant temperature at −24 °C during shipping is usually guaranteed when using temperature control media (phase change material). As a cheaper alternative, gel-based ice packs provide low temperatures only for a few hours. Afterward, the temperature is usually found to be around −0.5 °C, as shown in our experiments (Figure 2).

We also simulated poor laboratory practices by exposing the dry extracts to higher temperatures, such as leaving them in the refrigerator (+5 °C) or (in worse scenarios) on the bench (+23 °C) or a heated evaporator (+30 °C). The above-listed temperature conditions may also help better understand sample degradation and the extent of compromising the sample integrity.

After three days of storage, we resuspended the dry extracts in the particular solvents used for LC-MS assays (see Section 2.2) and analyzed them. We used untargeted metabolomics with hydrophilic interaction chromatography (HILIC) in positive and negative modes and reversed-phase liquid chromatography (RPLC) in negative mode with optimal conditions, as described before [18]. In addition, RPLC in positive and negative modes was used for untargeted lipidomics [18]. Examples of total ion chromatograms (TIC) for each LC-MS-based platform and extract type are shown in Figures S1 and S2. Furthermore, to increase the number of MS/MS spectra available for metabolite annotation, we employed an automated iterative exclusion method on selected samples [20]. Finally, we statistically evaluated the combined data sets using multivariate data analysis, followed by the Student’s *t*-test to analyze the differences between −80 °C (reference condition) and the rest of the storage temperatures.

### 3.1. Untargeted Metabolomics

Using three LC-MS-based untargeted metabolomics platforms, we annotated 146 endogenous metabolites and five exposome compounds (metformin, warfarin, caffeine, cotinine, and 3-hydroxycotinine) in human serum extracts (Table S1). The same LC-MS platforms also annotated 179 endogenous metabolites in mouse liver extracts (Table S2), including amino acids and their derivatives, dipeptides, biogenic amines, sugars, nucleotides, organic acids, and sugar phosphates.

**Figure 3:**
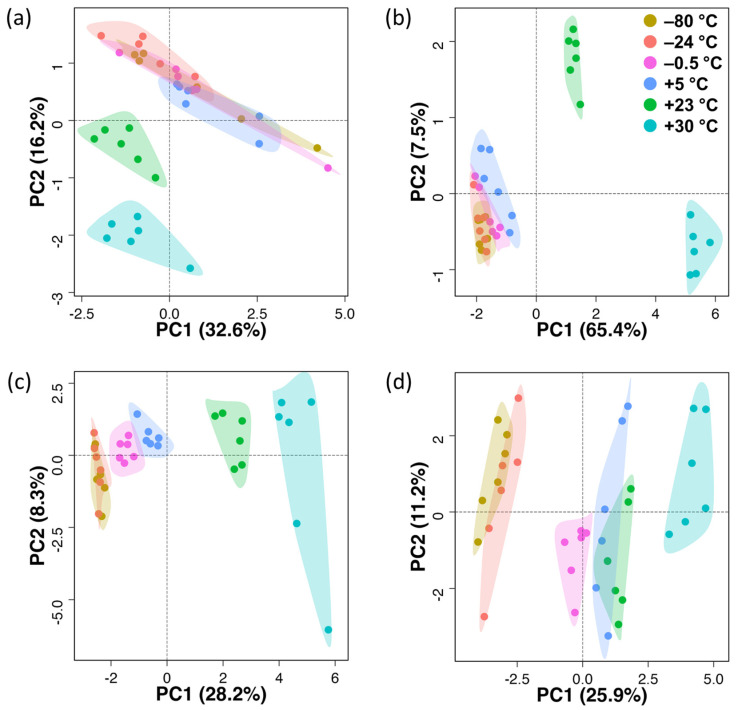
PCA scores plots for (**a**,**b**) metabolomics and (**c**,**d**) lipidomics data of (**a**,**c**) dry serum and (**b**,**d**) liver extracts at different storage temperatures. Labeling of data points according to storage temperature.

After quality control, we performed a principal component analysis (PCA) to obtain an overview of the gross variance of the data set (Figure 3). The PCA scores plots showed that different temperature storage conditions did not significantly impact the stability of dry serum extracts at low storage temperatures (Figure 3a). There was some overlap among the groups stored at −80 °C, −24 °C, −0.5 °C, and +5 °C, while the groups at +23 °C and +30 °C were separated. Statistical analysis of each polar metabolite using the Student’s *t*-test revealed that most polar metabolites in dry serum extracts were stable over three days at −24 °C, −0.5 °C, and +5 °C (Table 1). However, some metabolites, such as 5′-*S*-methyl-5′-thioadenosine and α-ketoglutaric acid, increased at high temperatures (+23 °C, +30 °C), while glucose, taurine, and uric acid decreased.

PCA scores plot for polar metabolites in dry liver extracts (Figure 3b) indicated overlapping clusters for −80 °C, −24 °C, and −0.5 °C groups, with the +5 °C group closely attached. However, the +23 °C and +30 °C groups were more separated from the other groups. The sum of the first and second principal components (PC1, PC2) combined accounted for 71.9% of the total variance in the dataset for dry liver extracts (Figure 3b) compared to 48.8% for dry serum extracts (Figure 3a).

Statistical analysis of each polar metabolite showed that polar metabolites in dry liver extracts were stable at −24 °C and −0.5 °C. Increasing the storage temperature to +5 °C impacted a few metabolites, such as 5′-*S*-methyl-5′-thioadenosine, pyridoxamine (increasing), *cis*-aconitic acid, and pentose phosphates (decreasing). However, higher temperatures significantly impacted metabolite stability (Table 1). Specifically, at +23 °C, 40 out of 179 metabolites showed significant changes compared to −80 °C, while at +30 °C, 90 out of 179 metabolites showed such changes in dry liver extracts. In most cases, a decrease in abundance was observed (e.g., dipeptides and amino acid derivatives) except for a few metabolites, such as 5′-*S*-methyl-5′-thioadenosine and citric acid, inosine 5′-monophosphate, *N*6,*N*6,*N*6-trimethyllysine, pyridoxamine, and methionine sulfoxide. These data show that the stability of polar metabolites in dry serum and liver extracts differed significantly between these two matrices and across different temperature storage conditions.

Enzymatic conversion, hydrolysis reactions, and oxidative processes may occur while storing biological samples and extracts. [24]. In addition, metabolomics extracts may contain residual proteins that were not completely removed during protein precipitation with organic solvents (methanol, MTBE) [25]. As a result, peptide bonds are susceptible to nonenzymatic hydrolysis and, therefore, might lead to formation of amino acid or peptide artifacts [24]. For instance, Wright et al. reported that deamination can convert asparagine and glutamine to their dicarboxylic acid counterparts, aspartate and glutamate [26]. However, we did not observe these reactions during the short-term stability. Furthermore, the decrease in the amount of the amino acid methionine in dry liver extracts may be explained by its oxidation to methionine sulfoxide [27], which increased with higher storage temperature in our study. Similarly, the decrease in *cis*-aconitic acid may be attributed to its thermal transformation to *cis*-aconitic anhydride [28].

In contrast, a large increase in 5′-*S*-methyl-5′-thioadenosine in dry serum and liver extracts with increasing storage temperatures revealed a likely cleavage of unstable *S*-adenosyl-L-methionine, a reaction that is accelerated above room temperature [29]. Additionally, an increase in creatinine levels can be explained as the breakdown of creatine [9]. However, this trend was observed only during the storage of dry liver extracts and not in serum extracts.

### 3.2. Untargeted Lipidomics

Using two LC-MS-based untargeted lipidomics platforms, we annotated 451 unique lipid species in human serum extracts covering 26 lipid subclasses (Table S1) and 545 unique lipid species covering 33 lipid subclasses in the mouse liver extracts (Table S2). Among them, lipids containing odd-chain fatty acids were also annotated (13% for serum and 16% for liver extracts from all lipids), although their signal intensity was lower than common lipids with even-chain fatty acids. Examples of annotated lipids belonging to different lipid classes containing odd-chain fatty acids using MS/MS and MS-DIAL software are shown in Figure S3.

In our initial exploration, a PCA scores plot for lipids in dry serum extracts (Figure 3c) showed overlapping clusters for the −80 °C and −24 °C groups, with closely clustered −0.5 °C and +5 °C groups, followed by separated +23 °C and +30 °C groups. The PCA scores plot for lipids in dry liver extracts (Figure 3d) showed also overlapping clusters for the −80 °C and −24 °C groups, followed by clustered −0.5 °C, +5 °C and +23 °C groups, and a more remote +30 °C group. The sum of PC1 and PC2 combined accounted for 36.5% of the total variance in the dataset for dry serum extracts (Figure 3c) and 37.2% of the total variance for dry liver extracts (Figure 3d). Overall, there was less variability observed in lipid composition after storage compared to polar metabolites.

Our separate statistical analysis of each lipid and matrix showed that many complex lipids in dry serum extracts were stable over three days at −24 °C, with slight changes in their abundances at −0.5 °C and +5 °C (Table 2). However, we observed more changes in the lipidome for both serum and liver extracts with increasing storage temperature. These changes mainly occurred in oxidized forms of fatty acids (oxFA), phosphatidylcholines (oxPC), phosphatidylinositols (oxPI), and triacylglycerols (oxTG).

**Figure 4:**
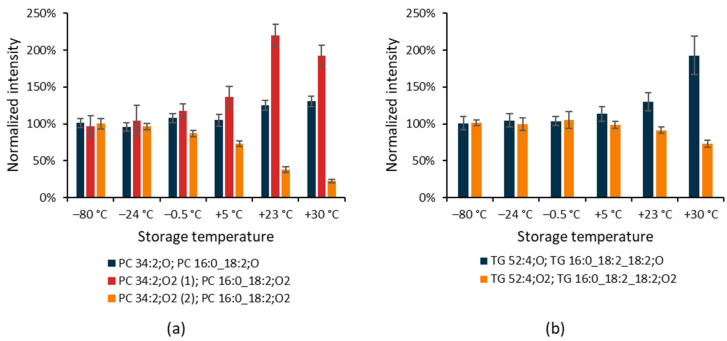
The impact of storage temperature on changes in oxidized PC and TG species in dry human serum extracts over three days. (**a**) PC 34:2;O (*m*/*z* 832.5709 at 1.16 min) and two positional isomers of PC 34:2:O2 detected as [M+CH_3_COO]^−^: (1) *m*/*z* 848.5658 at 1.07 min annotated as hydroxy or epoxide derivatives, (2) *m*/*z* 848.5658 at 1.16 min annotated as a hydroperoxide; (**b**) TG 52:4;O (*m*/*z* 888.7614 at 2.38 min) and TG 52:4;O2 (*m*/*z* 904.7557 at 2.35 min) detected as [M+NH_4_]^+^. Data are mean ± s.d. (*n* = 6).

In general, the double bonds in long carbon chains make the chemical integrity of lipid molecules vulnerable to oxidation [30]. For oxFA and oxPI, an increase in abundance with increasing storage temperature was apparent. For oxPC, we observed both increasing and decreasing trends. An increasing trend with storage temperature was found for oxPC species containing a single oxygen. Increasing and decreasing trends were detected for oxPC species containing two oxygens (Figure 4a), as shown in the example for PC 34:2;O, and two positional isomers of PC 34:2;O2 differing in their retention times. Using MS-DIAL [21], these two isomers were annotated as PC 16:0_18:2;O2, meaning that these species contain palmitic acid and oxidized linoleic acid with two oxygens which may represent different oxidative modifications (e.g., hydroxylation, hydroperoxide, peroxide, carboxylic acid, hydroxy-aldehyde) [31].

**Figure 5:**
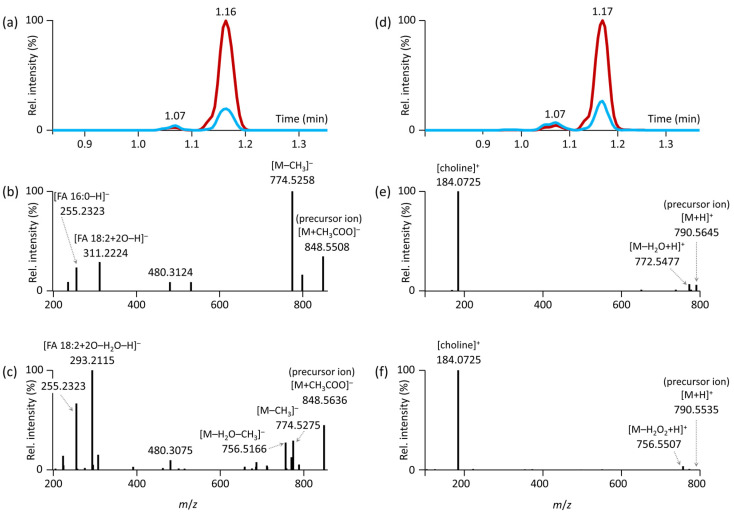
RPLC-MS/MS analysis and annotation of oxidized PC 16:0_18:2;O2 isomers. (**a**) Extracted ion chromatograms (EIC) in ESI(−) for the precursor ion *m*/*z* 848.5658, [M+CH_3_COO]^–^ in dry serum extracts (red EIC representing analysis of dry extract stored at −80 °C, blue EIC representing analysis of dry extract stored at +30 °C); (**b**) HR-MS/MS product ion spectrum of PC 16:0_18:2;O2 in ESI(−) eluted at 1.07 min; (**c**) HR-MS/MS product ion spectrum of PC 16:0_18:2;O2 in ESI(−) eluted at 1.16 min; (**d**) EIC in ESI(+) for the precursor ion *m*/*z* 790.5598, [M+H]^+^ in dry serum extracts (red EIC representing analysis of dry extract stored at −80 °C, blue EIC representing analysis of dry extract stored at +30 °C); (**e**) HR-MS/MS product ion spectrum of PC 16:0_18:2;O2 in ESI(+) eluted at 1.07 min; (**f**) HR-MS/MS product ion spectrum of PC 16:0_18:2;O2 in ESI(+) eluted at 1.17 min.

Deciphering the ESI(−)-MS/MS spectra, we found that a minor chromatographic peak *m*/*z* 848.5658 at 1.07 min (Figure 5a) contained the fragments *m*/*z* 255.2330 (palmitic acid) and *m*/*z* 311.2288 (oxidized linoleic acid as epoxide or hydroxy derivatives [32]) (Figure 5b). A second and more abundant chromatographic peak was detected at 1.16 min with the same precursor mass (Figure 5a). It contained the fragment ions *m*/*z* 255.2330 (palmitic acid), but also a fragment *m*/*z* 293.2122 supporting the presence of hydroperoxide derivatives [33] of linoleic acid after H_2_O loss (Figure 5c). A similar mechanism was also described during the MS/MS fragmentation of hydroperoxides of triacylglycerols [34]. The fragment [M–CH_3_]^–^, typically observed for phosphatidylcholines, dominated for PC 34:2;O2 (*m*/*z* 774.5258, Figure 5b) with the presence of epoxide or hydroxy derivatives, while its intensity was much lower in PC 34:2;O2 with the presence of hydroperoxide derivatives (Figure 5c).

These findings were further supported by the ESI(+)-MS/MS spectra. The corresponding protonated molecules (Figure 5d) of the minor peak provided a fragment ion due to H_2_O loss, supporting the presence of epoxide or hydroxy derivatives. In contrast, the major chromatographic peak showed a fragment ion due to the loss of H_2_O_2_, supporting the presence of hydroperoxide derivatives (Figure 5f). These observations are consistent with a recent study on oxidized phosphatidylcholines [33]. Oxidized TG annotated in dry serum extracts exhibited an increasing trend with storage temperature for species containing a single oxygen and a decreasing trend for species with two oxygens in the molecule, as shown in Figure 4b on an example for TG 52:4;O and TG 52:4;O2.

**Figure 6:**
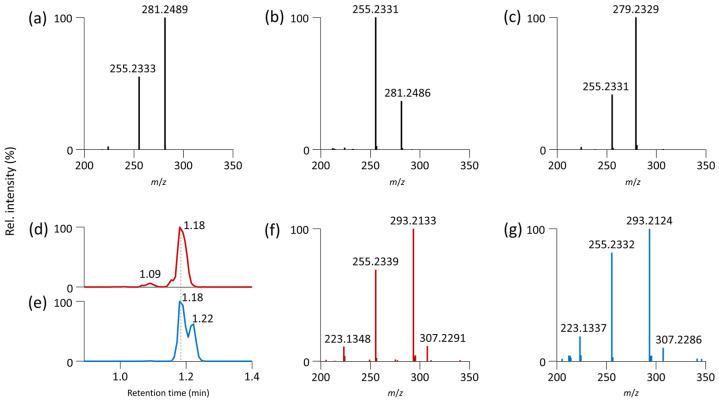
RPLC-MS/MS analysis for confirmation of regioisomers. ESI(−)-MS/MS spectrum (zoom of *m*/*z* 200–350) of (**a**) PC 16:0/18:1 standard (precursor ion *m*/*z* 818.5917, [M+CH_3_COO]^–^, retention time 1.76 min); (**b**) PC 18:1/16:0 standard (precursor ion *m*/*z* 818.5917, [M+CH_3_COO]^–^, retention time 1.76 min); (**c**) PC 16:0/18:2 standard (precursor ion *m*/*z* 816.5760, [M+CH_3_COO]^–^, retention time 1.62 min); EIC of (**d**) PC 16:0/18:2;OOH in serum extract (*m*/*z* 848.5658, retention time 1.18 min); and (**e**) PC 16:0/18:2;OOH (*m*/*z* 848.5658, [M+CH_3_COO]^–^, retention time 1.18 min) formed by oxidation of the PC 16:0/18:2 standard; ESI(−)-MS/MS spectrum (zoom of *m*/*z* 200–350) of (**f**) PC 16:0/18:2;OOH in serum extract (precursor ion *m*/*z* 848.5658, [M+CH_3_COO]^–^); (**g**) PC 16:0/18:2;OOH formed by oxidation of the PC 16:0/18:2 standard (precursor ion *m*/*z* 848.5658, [M+CH_3_COO]^–^).

In the next step, we attempted to elucidate the regioisomeric composition of oxidized PC. Analysis of commercially available (non-oxidized) PC standards was used to confirm fragmentation behavior. For PC 16:0/18:1, the 18:1 ion (*m*/*z* 281.2489) was the most abundant, while the 16:0 ion (*m*/*z* 255.2333) had an intensity of around 50% of the 18:1 ion (Figure 6a). The ratios of fatty acid ions were reversed for the other regioisomer PC 18:1/16:0, indicating that the fatty acid is more easily cleaved from the *sn*-2 position (Figure 6b). For PC 16:0/18:2, a derived form of oxidized PC 16:0_18:2;O2, the 18:2 ion (*m*/*z* 279.2329) was the most abundant (Figure 6c). Although the PC 18:2/16:0 standard was unavailable, Fabritius and Yang reported reversed fragmentation patterns for PC 18:1/16:0 [35]. Since individual oxidized forms of PC 16:0_18:2 are not commercially available, we submitted the PC 16:0/18:2 standard to oxidation (heating at 50 °C for 24 h) to obtain residues of its oxidized form. Follow-up analysis of the serum extract (Figure 6d,f) and oxidized standard (Figure 6e,g) showed that PC 16:0/18:2;OOH had the same retention time and MS/MS spectra. The [FA 18:2+2O–H_2_O–H]^–^ ion (*m*/*z* 293.2133) was the most abundant, and the 16:0 ion (*m*/*z* 255.2339) had an intensity of 80–90%.

However, the PC 18:0/20:4 standard provided an intensive 20:4 ion (*m*/*z* 303.2329) and a less abundant 18:0 ion (*m*/*z* 283.2643) (Figure S4a), while the oxidized form PC 18:0/20:4;O had an intensive ion corresponding to 18:0 (*m*/*z* 283.2643) and a less abundant hydroxy FA 20:4 (*m*/*z* 319.2291) (Figure S4b). This finding contradicts previous observations that fragments of fatty acids from the *sn*-2 position are more intense. Thus, determining the regioisomeric composition of oxidized PC based on the ratios of fatty acid fragments in negative ion mode is more challenging than for non-oxidized forms, due to factors such as the number of double bonds and oxygens involved, as well as the position of oxidation within the fatty acid chain.

Interestingly, FA 18:3;O and two isomers of FA 22:6;O were stable in dry serum during all storage conditions, while in the liver extracts, their intensities increased even at −0.5 °C storage temperature. Contrarily, oxFA 20:3 and 20:4 were stable in the liver extracts and increased in dry serum extracts with increasing temperature. However, targeted LC-MS/MS analysis or different fragmentation mechanisms, such as electron-activated dissociation, would be needed to clarify the structure of these compounds [36]. In addition, the lyso-forms of PI (LPI) increased with the storage temperature in dry serum extracts, while the lyso-forms of phosphatidylethanolamines (LPE) decreased with the storage temperature in dry serum extracts but increased in dry liver extracts. A few ether-linked TG also decreased in dry serum extracts with increasing temperature during storage. Nonetheless, the lipidome of dry liver extracts was less altered compared to dry serum extracts. Importantly, the main changes in the lipidome of serum and liver extracts were related to already present oxidized forms of various lipid classes. Furthermore, the number of annotated oxidized lipids in the serum (*n* = 50) used in this study was higher compared to the liver (*n* = 34) extracts.

Finally, we summarized the results of this study in Table 3, highlighting polar metabolites and lipid subclasses that were mainly impacted during short-term stability.

## 4. Conclusions

In this study, we assessed the stability of polar metabolites and complex lipids in dry human serum and mouse liver extracts over a period of three days under different temperature conditions (−80 °C, −24 °C, −0.5 °C, +5 °C, +23 °C, and +30 °C). These temperatures were chosen to simulate the time between sample extraction and analysis, as well as optional shipping of dry extracts to different laboratories, and to determine how higher temperatures affect sample integrity. Using five fast LC-MS methods followed by data processing, over 600 metabolites were statistically evaluated. Our findings showed that storage of dry extracts at −24 °C and partially at −0.5 °C yielded comparable results to the reference condition of −80 °C. However, increasing storage temperatures led to significant changes in polar metabolites and oxidized lipids such as oxTG, oxPC, oxPE, oxPI, and oxFA within three days.

## Figures and Tables

**Table 1:** The percentage of polar metabolites statistically differed during the short-term storage of dry serum and liver extracts for three days at different storage conditions compared to −80 °C (reference condition).

Temperature	Storage	Serum	Liver
−24 °C	freezer	0.0%	0.0%
−0.5 °C	box with ice packs	0.0%	0.6%
+5 °C	refrigerator	0.0%	2.8%
+23 °C	laboratory	2.6%	22.2%
+30 °C	thermostat	3.3%	50.0%

**Table 2:** The percentage of complex lipids statistically differed during the short-term storage of dry serum and liver extracts for three days at different storage conditions compared to −80 °C (reference condition).

Temperature	Storage	Serum	Liver
−24 °C	freezer	0.0%	0.0%
−0.5 °C	box with ice packs	0.9%	2.6%
+5 °C	refrigerator	2.4%	2.6%
+23 °C	laboratory	8.8%	4.2%
+30 °C	thermostat	13.9%	5.9%

**Table 3:** Overview of trends of metabolites during the short-term storage of dry serum and liver extracts with increasing storage temperature compared to −80 °C (reference condition).

Trend	Dry Serum Extracts	Dry Liver Extracts
Increasing	oxFA (O; O2; O3), LPI, oxPC (O; O2), oxPI (O), oxTG (O), FA, LPC, 5′-*S*-methyl-5′-thioadenosine, α-ketoglutaric acid	oxFA (O), LPE, oxPC (O; O2), oxPE (O2), oxPI (O; O2), 5′-*S*-methyl-5′-thioadenosine, arginine, creatinine, pyridoxamine, *N*-acetylphenylalanine, *N*-ε-dimethyllysine, citric acid, inosine 5′-monophosphate, methioninesulfoxide, *N*6,*N*6,*N*6-trimethyllysine
Decreasing	LPE, oxPC (O2), PE, etherPE, oxTG (O2), glucose, taurine, uric acid	ether-TG, 1-/3-methylhistidine, acetylcholine, *cis*-aconitic acid, adenosine, dipeptides, citrulline, histidine, hypotaurine, pentose phosphates, hexose phosphates, TMAO, pyridoxal 5′-phosphate, ornithine, *N*-acetylhistidine, *N*-α-acetyllysine, cystathionine, glutamic acid, glutamine, methionine

## Data Availability

Data are contained within this article and Supplementary Materials.

## Supplementary Materials

The following supporting information can be downloaded at: https://www.mdpi.com/article/10.3390/antiox12050986/s1, Table S1. Metabolomics and lipidomics data for human serum extracts; Table S2. Metabolomics and lipidomics data for mouse liver extracts; Table S3. Lipid classes in MS-DIAL used for annotation; Figure S1. Examples of total ion chromatograms for human serum extracts acquired using LC-MS methods; Figure S2. Examples of total ion chromatograms for mouse liver extracts acquired using LC-MS methods; Figure S3. Examples of annotated lipids containing odd-chain fatty acids using MS/MS and MS-DIAL software; Figure S4. ESI(−)-MS/MS spectrum of PC 18:0/20:4 and PC 18:0/20:4;O.
