# Supplementary material for: Short-Term Stability of Serum and Liver Extracts for Untargeted Metabolomics and Lipidomics
Source: Antioxidants (Basel). 2023 Apr 24;12(5):986. doi: 10.3390/antiox12050986 (PMC10215277; doi:10.3390/antiox12050986)
Supplement: Supplementary file 1 [file antioxidants-12-00986-s001.zip › antioxidants-2340912_Supplementary_Materials_Figures_S1-S4.pdf]

Supplementary Materials

Short-Term Stability of Serum and Liver Extracts for Untargeted Metabolomics and Lipidomics

Jiri Hricko [1], Lucie Rudl Kulhava [1], Michaela Paucova [1], Michaela Novakova [1], Ondrej Kuda [1], Oliver Fiehn [1] and Tomas Cajka [1,*]

[1] Institute of Physiology of the Czech Academy of Sciences, Videnska 1083, 14200 Prague, Czech Republic

[2] West Coast Metabolomics Center, University of California, Davis, 451 Health Sciences Drive, Davis, CA, 95616, USA

* Correspondence: tomas.cajka@fgu.cas.cz

Figure S1. Examples of total ion chromatograms for human serum extracts acquired using LC-MS methods: (**a**) RPLC-ESI(+) lipidomics (ACQUITY Premier BEH C18 column); (**b**) RPLC-ESI(–) lipidomics (ACQUITY Premier BEH C18 column); (**c**) HILIC-ESI(+) metabolomics (BEH Amide column); (**d**) HILIC-ESI(–) metabolomics (ACQUITY Premier BEH Amide column); (**e**) RPLC-ESI(–) metabolomics (ACQUITY Premier HSS T3 column).

Figure S2. Examples of total ion chromatograms for mouse liver extracts acquired using LC-MS methods: (**a**) RPLC-ESI(+) lipidomics (ACQUITY Premier BEH C18 column); (**b**) RPLC-ESI(–) lipidomics (ACQUITY Premier BEH C18 column); (**c**) HILIC-ESI(+) metabolomics (BEH Amide column); (**d**) HILIC-ESI(–) metabolomics (ACQUITY Premier BEH Amide column); (**e**) RPLC-ESI(–) metabolomics (ACQUITY Premier HSS T3 column).

Figure S3. Examples of annotated lipids containing odd-chain fatty acids using MS/MS and MS-DIAL software

RPLC-ESI(+)

- DG 35:2|DG 17:0_18:2
- LPC 17:0/0:0
- PC 35:2|PC 17:0_18:2
- SM 35:1;O2|SM 18:1;O2/17:0
- TG 51:1|TG 16:0_17:0_18:1

RPLC-ESI(–)

- CL 71:7|CL 17:1_18:2_18:2_18:2
- LPE 17:0
- PE 35:2|PE 17:0_18:2
- PE O-37:5|PE O-17:1_20:4
- PI 39:4|PI 19:0_20:4
- PS 37:4|PS 17:0_20:4

Figure S4. ESI(–)-MS/MS spectrum (zoom of *m*/*z* 200–350) of (**a**) the PC 18:0/20:4 standard (precursor ion *m*/*z* 868.6073, $[M+CH_3COO]^-$, retention time 1.76 min); (**b**) PC 18:0/20:4;O (precursor ion *m*/*z* 884.6021, $[M+CH_3COO]^-$, retention time 1.50 min) formed by oxidation of the PC 18:0/20:4 standard.

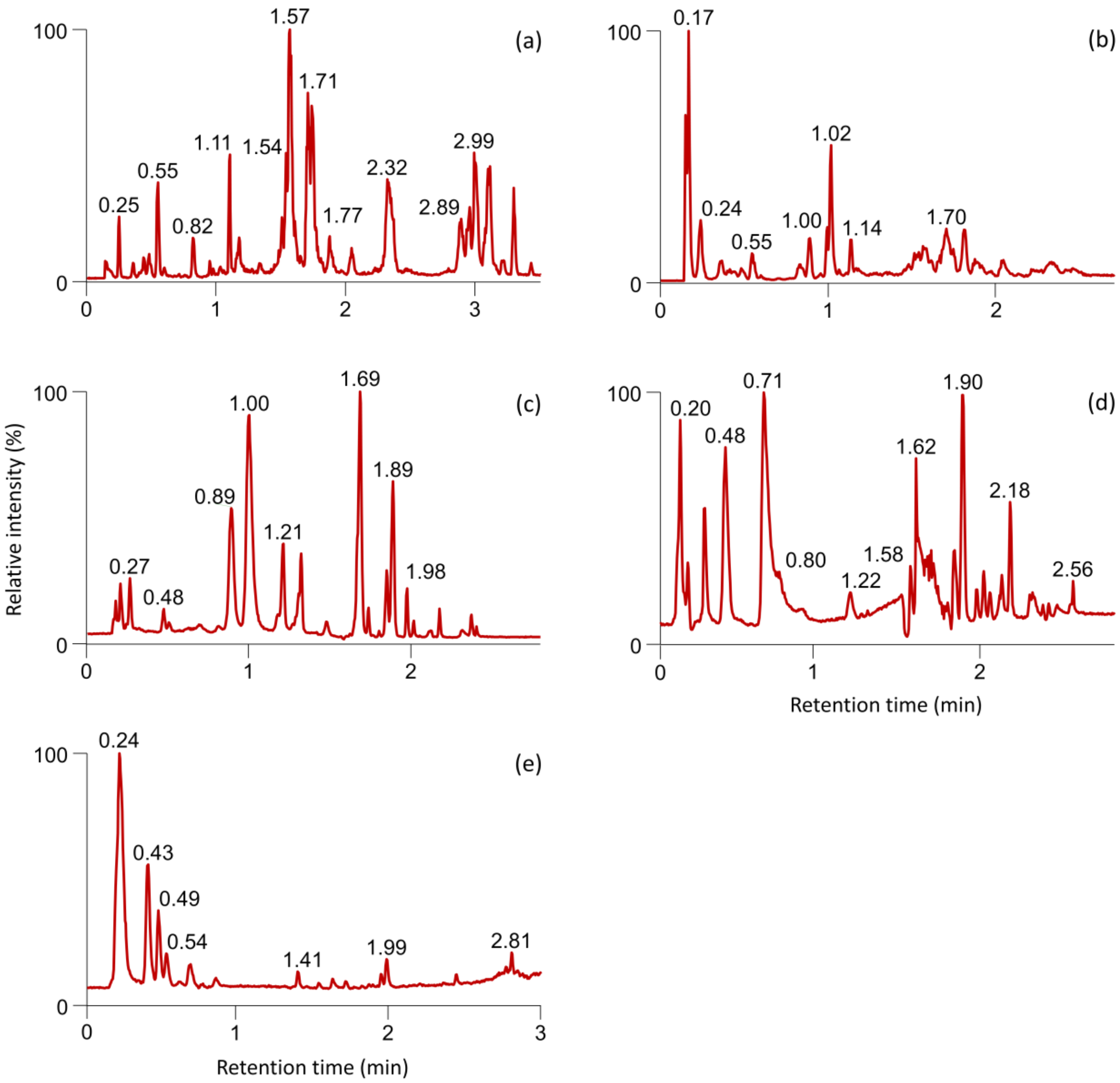

Figure S1. Examples of total ion chromatograms for human serum extracts acquired using LC-MS methods: (**a**) RPLC-ESI(+) lipidomics (ACQUITY Premier BEH C18 column); (**b**) RPLC-ESI(–) lipidomics (ACQUITY Premier BEH C18 column); (**c**) HILIC-ESI(+) metabolomics (BEH Amide column); (**d**) HILIC-ESI(–) metabolomics (ACQUITY Premier BEH Amide column); (**e**) RPLC-ESI(–) metabolomics (ACQUITY Premier HSS T3 column).

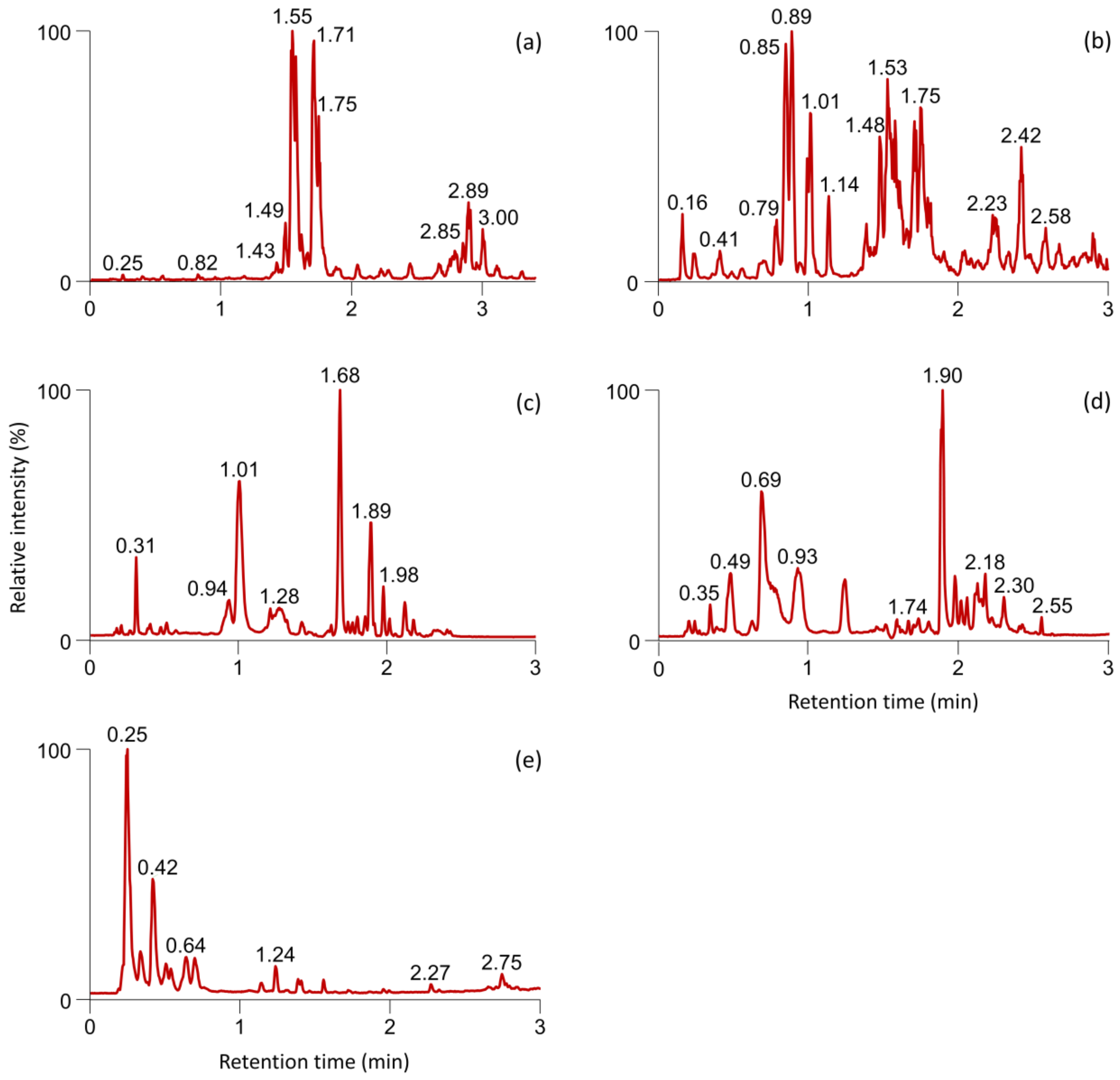

Figure S2. Examples of total ion chromatograms for mouse liver extracts acquired using LC-MS methods: (**a**) RPLC-ESI(+) lipidomics (ACQUITY Premier BEH C18 column); (**b**) RPLC-ESI(–) lipidomics (ACQUITY Premier BEH C18 column); (**c**) HILIC-ESI(+) metabolomics (BEH Amide column); (**d**) HILIC-ESI(–) metabolomics (ACQUITY Premier BEH Amide column); (**e**) RPLC-ESI(–) metabolomics (ACQUITY Premier HSS T3 column).

Figure S3. Examples of annotated lipids containing odd-chain fatty acids using MS/MS and MS-DIAL software

DG 35:2|DG 17:0_18:2

Retention time:	1.99 min
Ion form:	$[M+NH_4]^+$
Precursor ion:	*m*/*z* 624.55353 (ref. *m*/*z* 624.55621)

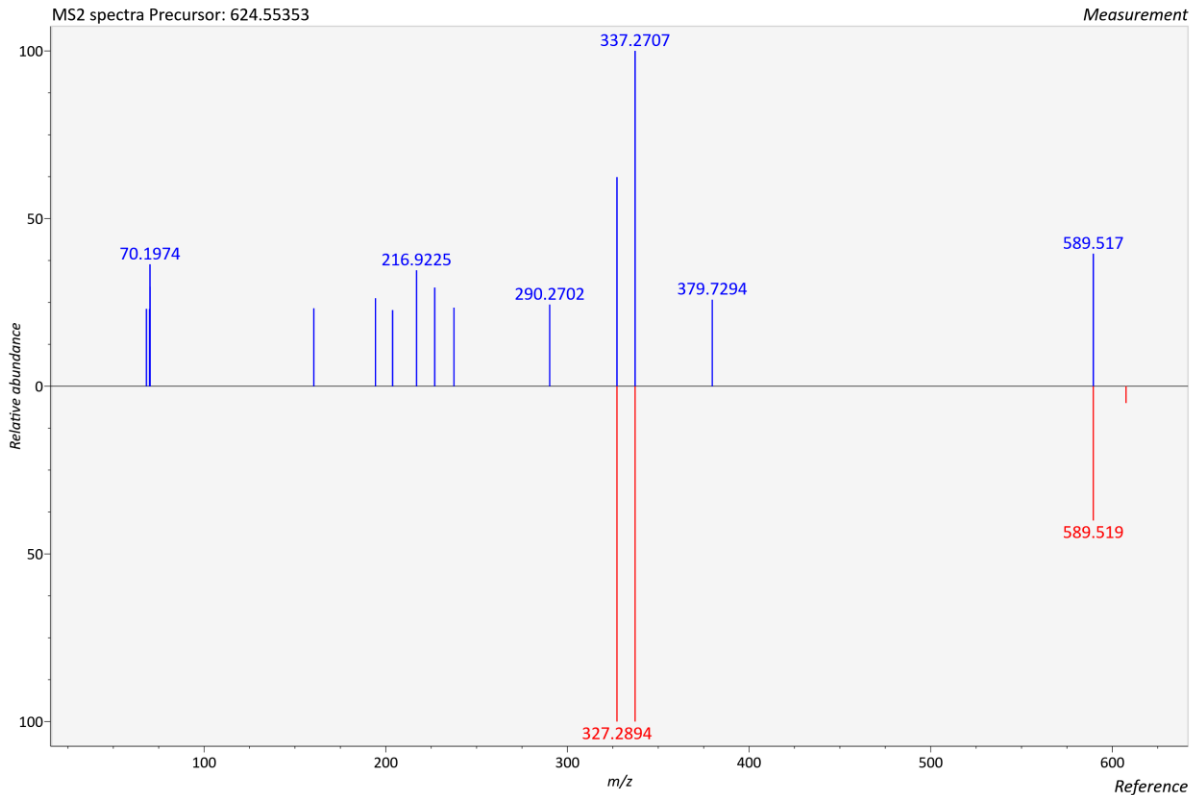

LPC 17:0/0:0

Retention time:	0.69 min
Ion form:	$[M+H]^+$
Precursor ion:	*m*/*z* 510.35373 (ref. *m*/*z* 510.35541)

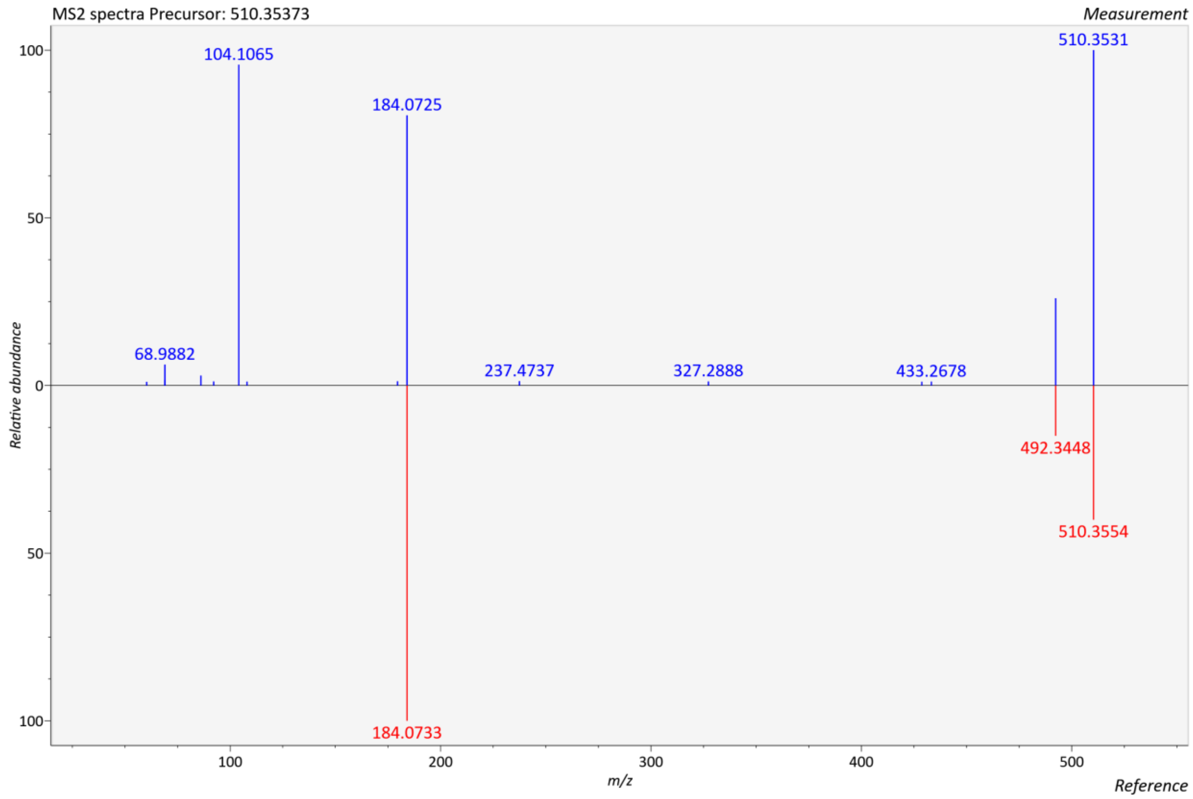

PC 35:2|PC 17:0_18:2

Retention time: 1.66 min

Ion form: $[M+H]^+$

Precursor ion: *m/z* 772.58154 (ref. *m/z* 772.58508)

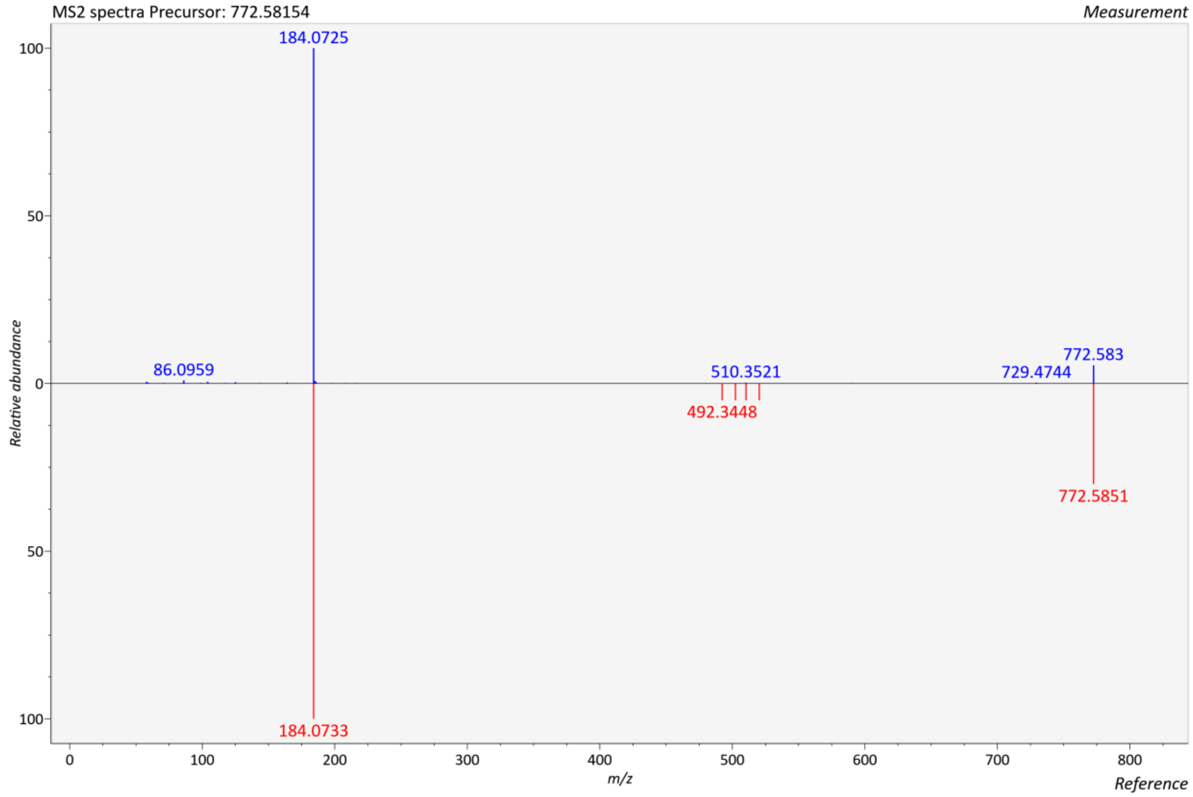

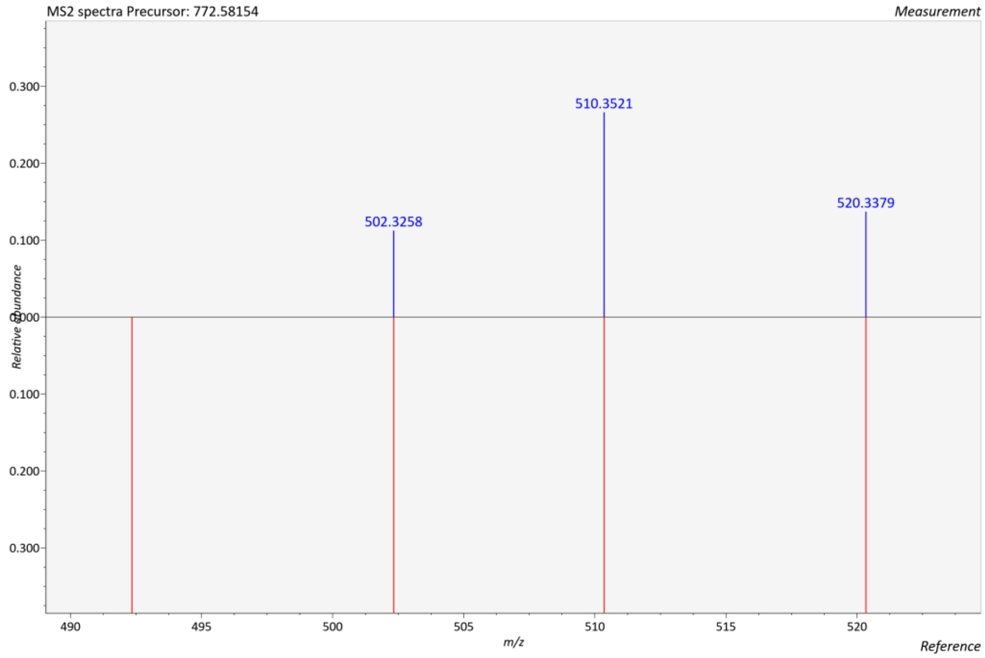

SM 35:1;O2 | SM 18:1;O2/17:0

Retention time:	1.60 min
Ion form:	$[M+H]^+$
Precursor ion:	*m*/*z* 717.58740 (ref. *m*/*z* 717.59052)

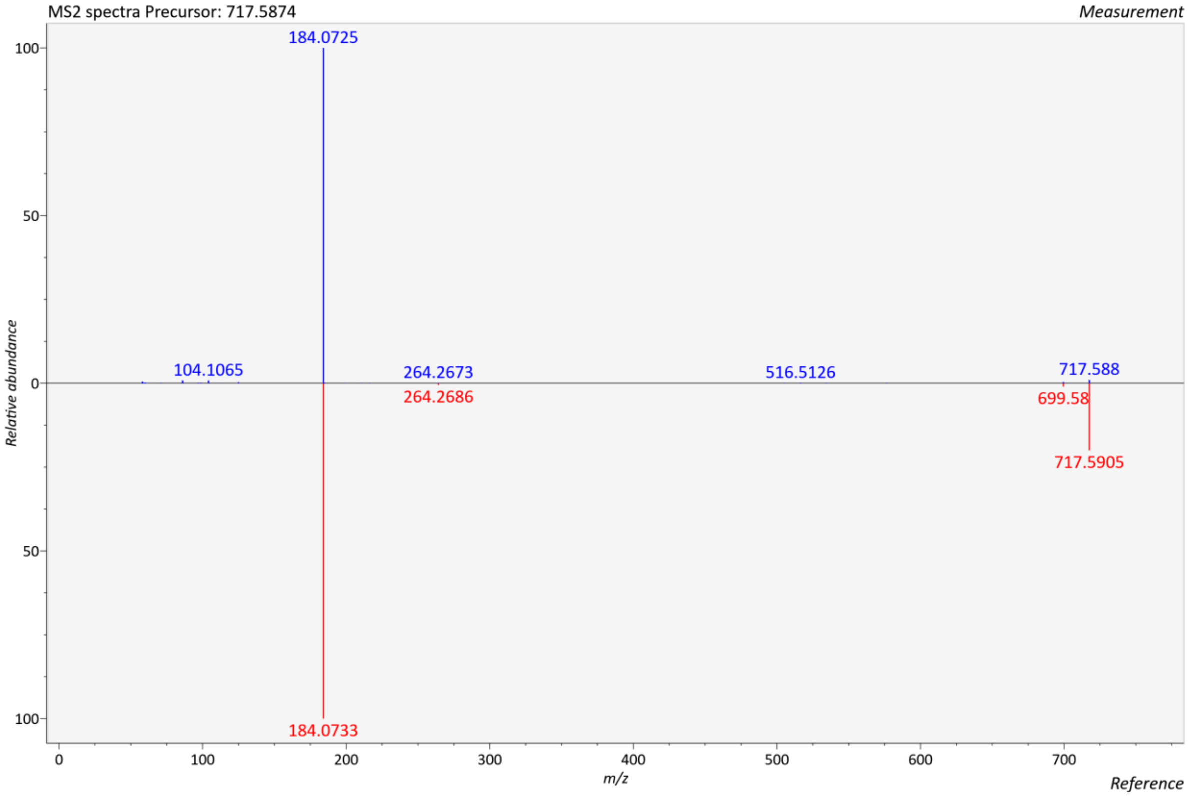

TG 51:1 | TG 16:0_17:0_18:1

Retention time: 3.16 min

Ion form: $[M+NH_4]^+$

Precursor ion: *m/z* 864.80072 (ref. *m/z* 864.80151)

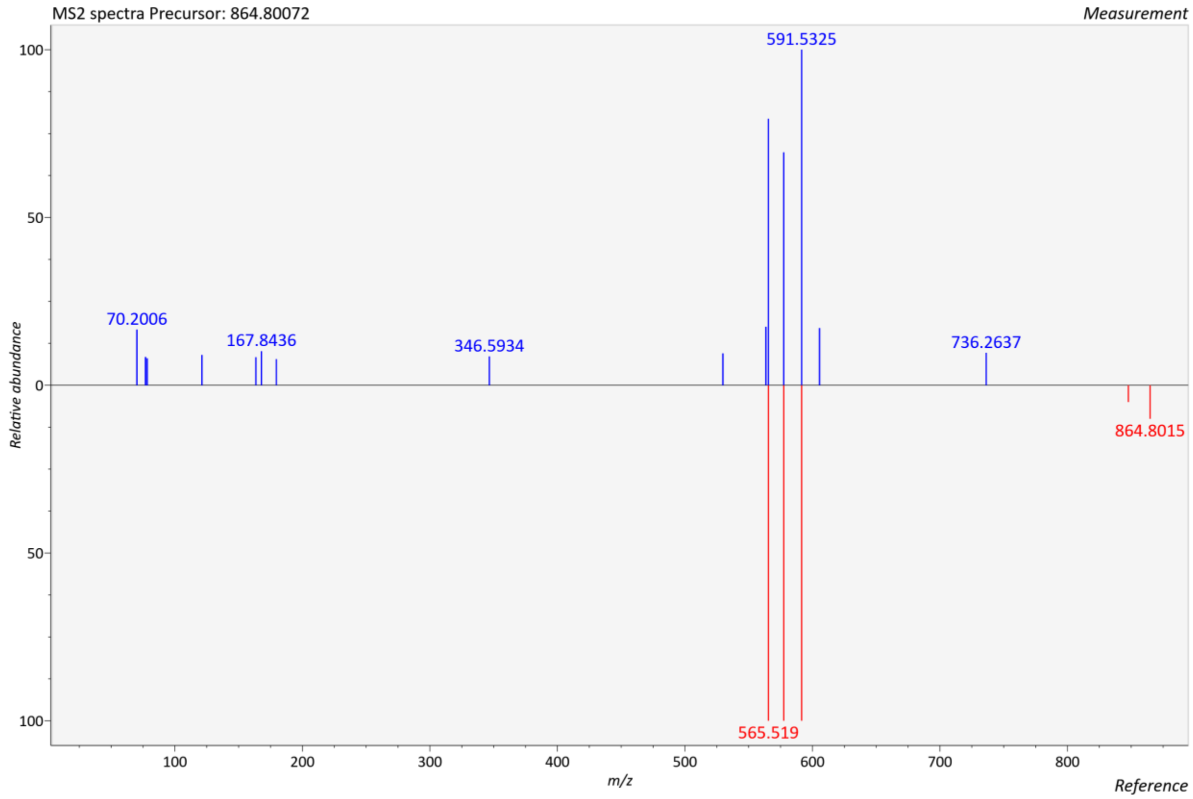

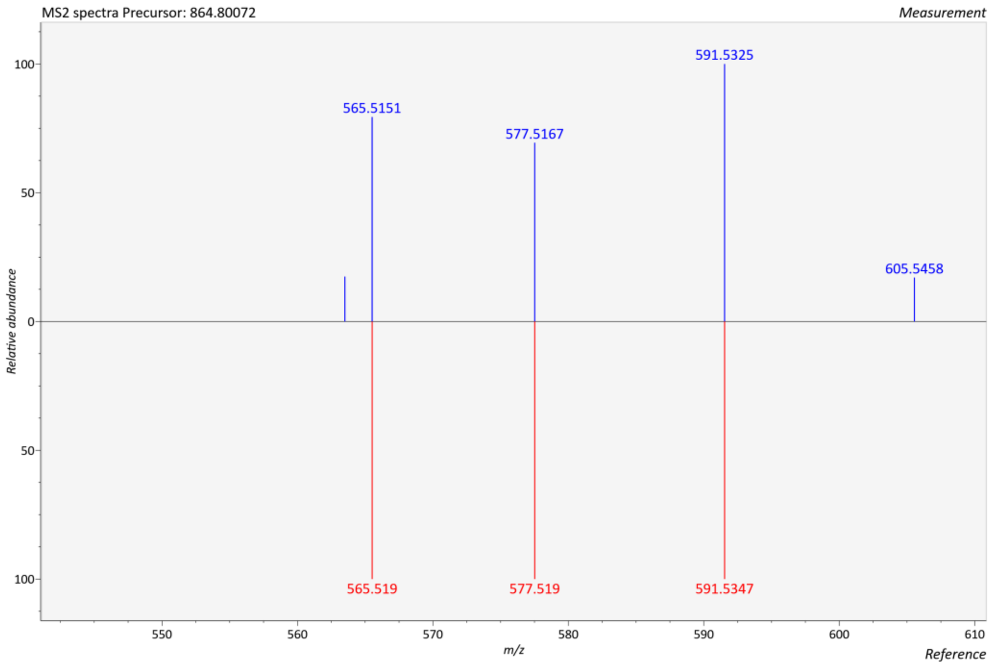

CL 71:7 | CL 17:1_18:2_18:2_18:2

Retention time: 2.62 min

Ion form: $[M–H]^-$

Precursor ion: *m*/*z* 1435.96289 (ref. *m*/*z* 1435.96484)

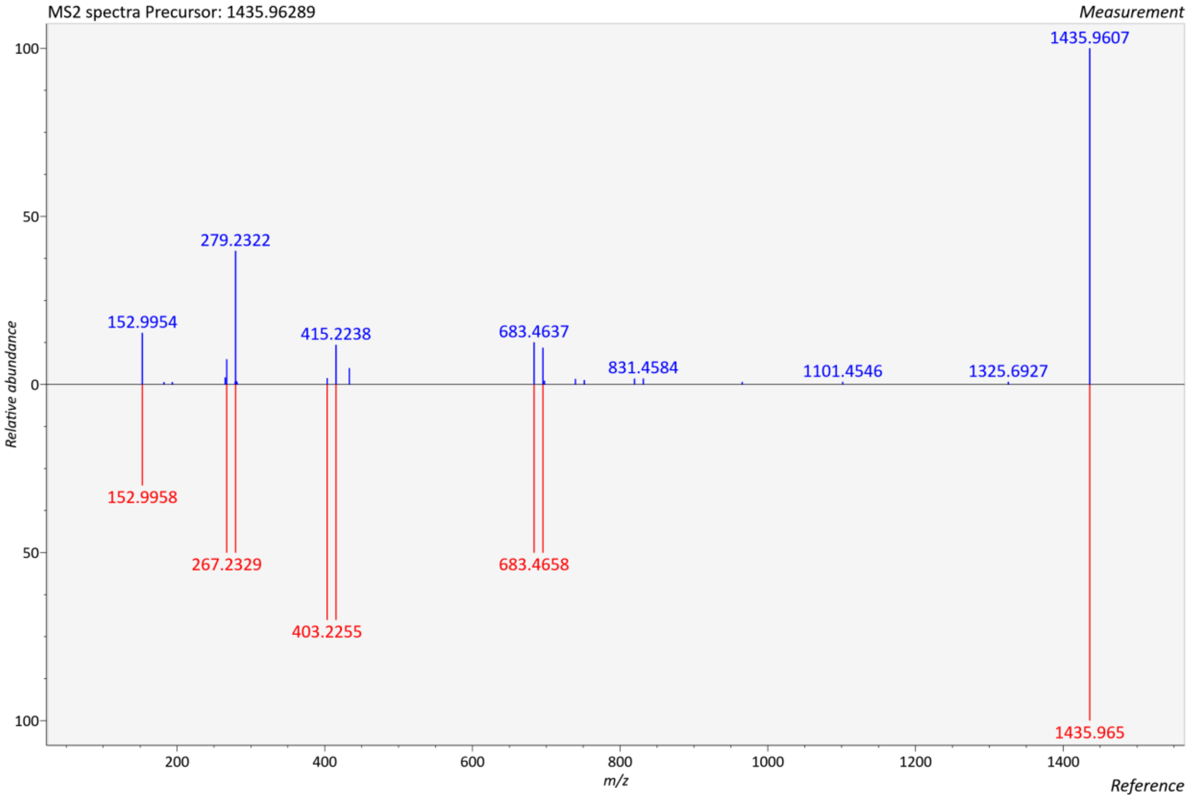

LPE 17:0

Retention time: 0.70 min

Ion form: [M–H]⁻

Precursor ion: *m/z* 466.29227 (ref. *m/z* 466.29391)

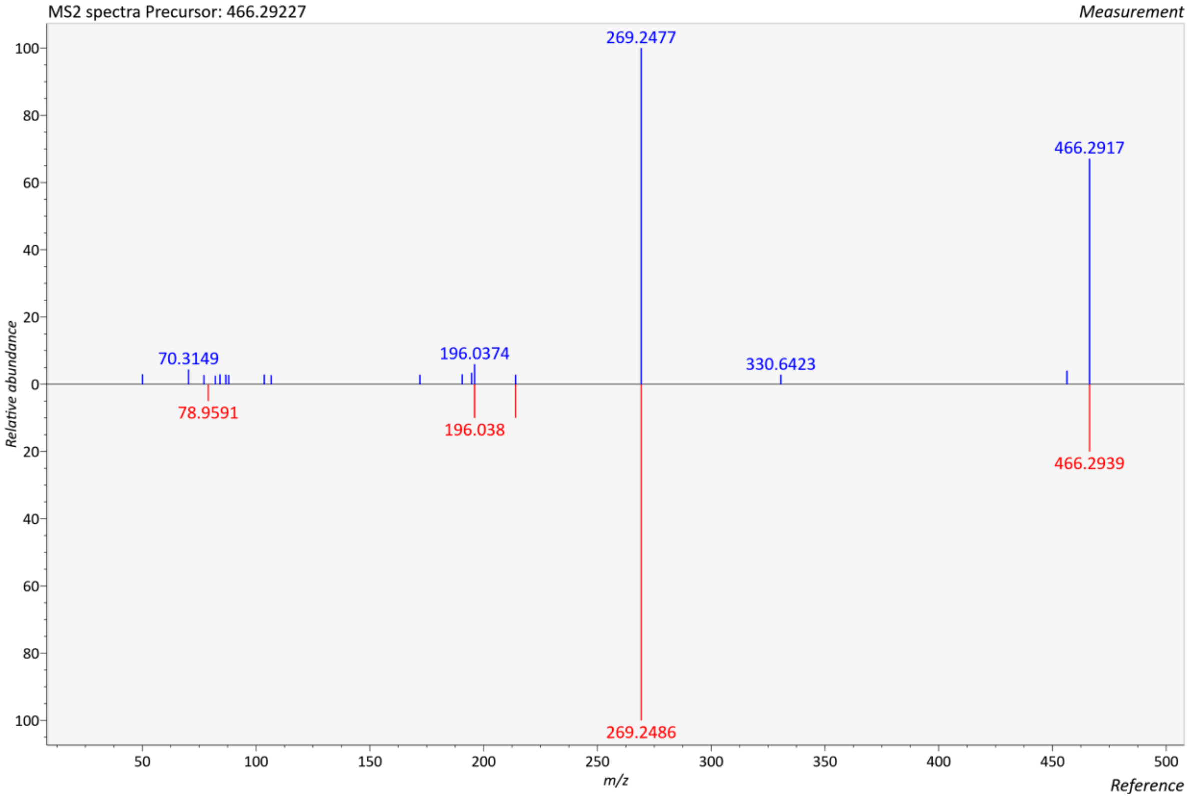

PE 35:2 | PE 17:0_18:2

Retention time:	1.71 min
Ion form:	[M–H]⁻
Precursor ion:	*m/z* 728.52222 (ref. *m/z* 728.52362)

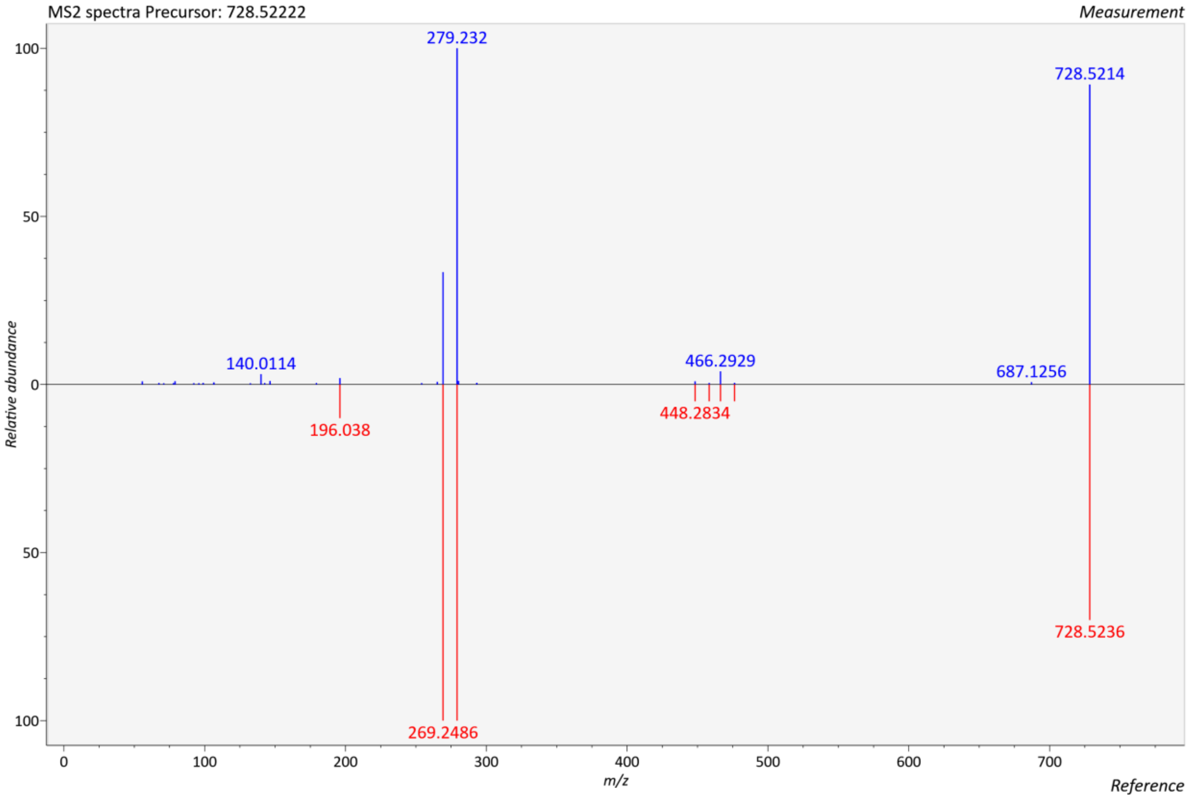

PE O-37:5 | PE O-17:1_20:4

Retention time:	1.72 min
Ion form:	[M–H]⁻
Precursor ion:	*m/z* 736.52789 (ref. *m/z* 736.52869)

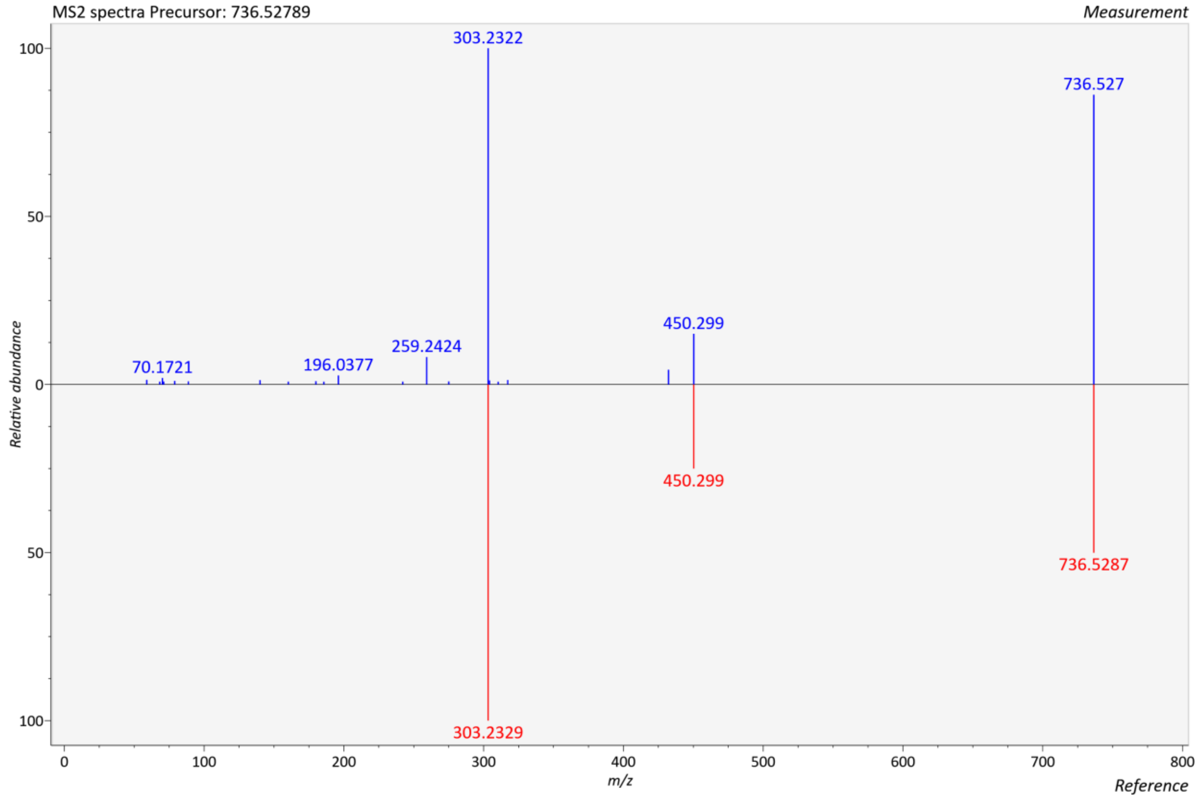

PI 39:4 | PI 19:0_20:4

Retention time:	1.61 min
Ion form:	[M–H]⁻
Precursor ion:	*m/z* 899.56213 (ref. *m/z* 899.56549)

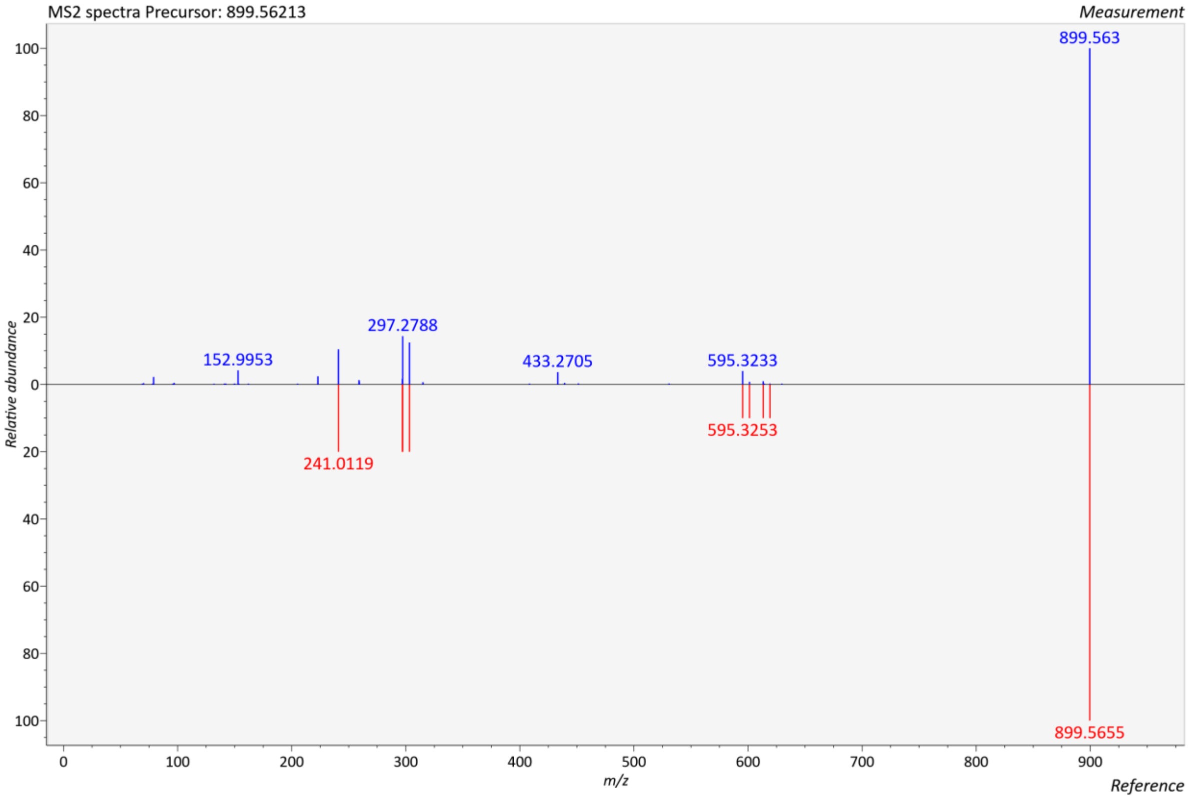

PS 37:4|PS 17:0_20:4

Retention time: 1.48 min

Ion form: [M–H]⁻

Precursor ion: *m*/*z* 796.51184 (ref. *m*/*z* 796.51343)

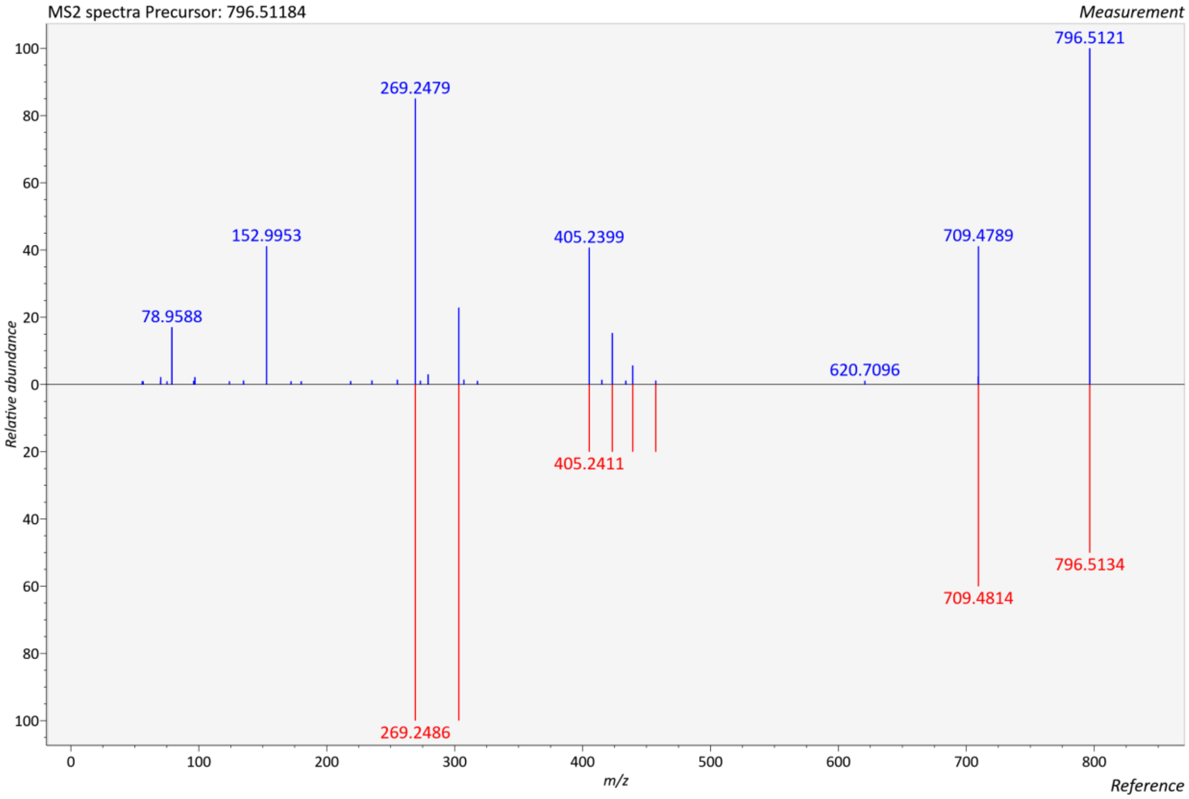

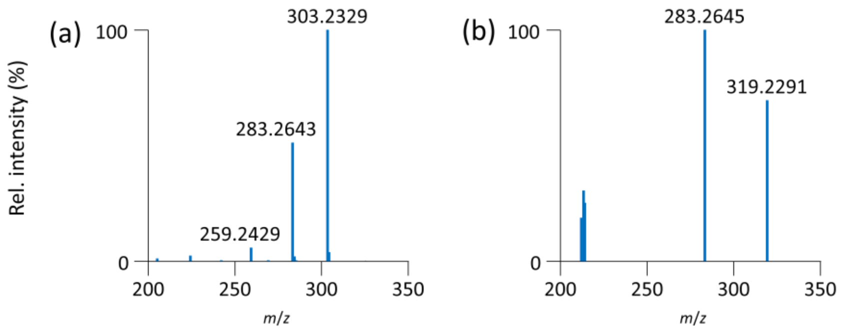

Figure S4. ESI(–)-MS/MS spectrum (zoom of *m*/*z* 200–350) of (**a**) the PC 18:0/20:4 standard (precursor ion *m*/*z* 868.6073, $[M+CH_3COO]^-$, retention time 1.76 min); (**b**) PC 18:0/20:4;O (precursor ion *m*/*z* 884.6021, $[M+CH_3COO]^-$, retention time 1.50 min) formed by oxidation of the PC 18:0/20:4 standard.
